# Supplementary material for: Exploring the role of neuronal-enriched extracellular vesicle miR-93 and interoception in major depressive disorder
Source: Transl Psychiatry. 2024 Apr 27;14:199. doi: 10.1038/s41398-024-02907-x (PMC11055873; doi:10.1038/s41398-024-02907-x)
Supplement: Supplementary file 1 — Supplemental Materials [file 41398_2024_2907_MOESM1_ESM.docx]

**Supplemental Materials**

**Exploring the role of neuronal-enriched extracellular vesicle miR-93 and interoception in major depressive disorder**

Kaiping Burrows, Ph.D.^1#*^, Leandra K. Figueroa-Hall, Ph.D.^1,2#^, Jennifer L. Stewart, Ph.D.^1,2^, Ahlam M. Alarbi, Ph.D.^3^, Rayus Kuplicki, Ph.D.^1^, Bethany N. Hannafon, Ph.D.^4^, Chibing Tan, Ph.D.^3^, Victoria B. Risbrough, Ph.D.^5,6^, Brett A. McKinney, Ph.D.^7^, Rajagopal Ramesh, Ph.D.^8^, Teresa A. Victor, Ph.D.^1^, Robin Aupperle, Ph.D.^1,2^, Jonathan B. Savitz, Ph.D.^1,2^, T. Kent Teague, Ph.D.^3,9,10^, Sahib S. Khalsa, M.D. ^1,2^, & Martin P. Paulus M.D.^1,2^

1. Laureate Institute for Brain Research, Tulsa, OK, USA.

2. Department of Community Medicine, University of Tulsa, Tulsa, OK, USA.

3. Departments of Surgery and Psychiatry, School of Community Medicine, The University of Oklahoma, Tulsa, OK, USA.

4. Department of Obstetrics and Gynecology, University of Oklahoma Health Sciences Center.

5. Center of Excellence for Stress and Mental Health, La Jolla, CA, USA.

6. Department of Psychiatry, University of California, San Diego, La Jolla, CA, USA.
7. Department of Mathematics and Computer Science, University of Tulsa, Tulsa, OK, USA.

8. Department of Pathology, University of Oklahoma Health Sciences Center, Oklahoma City, OK, USA.

9. Department of Biochemistry and Microbiology, The Oklahoma State University Center for Health Sciences, Tulsa, OK, USA.

10. Department of Pharmaceutical Sciences, The University of Oklahoma College of Pharmacy, Oklahoma City, OK, USA.

^#^ Kaiping Burrows and Leandra K. Figueroa-Hall should be considered joint first author.

* Corresponding author: Kaiping Burrows, Ph.D., Laureate Institute for Brain Research, 6655 South Yale Ave, Tulsa, OK. 74136; Phone: 918-502-5117; Fax: 918-502-5113; Email: [kburrows@laureateinstitute.org](mailto:kburrows@laureateinstitute.org)

**Supplemental Methods**

**2.2 Neuronal-enriched EV**

**2.2.3 NEEV enrichment.** EVs were enriched via a magnetic streptavidin bead immunocapture kit targeting the neural adhesion marker, L1CAM (CD171) biotinylated antibody, see *Burrows et al.* ^1^ Figure 7A for schematic carton of the NEEV enrichment. This technology for enriching NEEVs in blood samples has been previously validated ^1-4^. The CD171 (L1CAM, neural adhesion protein) marker was used for NEEV enrichment due to its high and relatively specific expression in neurons and low levels of expression in many other cell types ^2^. Briefly, 20 μL of 100 nanograms/μL of mouse anti-human CD171 biotinylated antibody (clone 5G3, eBioscience, United States; Catalog #13-1719-82) was incubated with 80 μL of 9.1 μm diameter covalently cross-linked streptavidin magnetic beads (System Biosciences, CA, United States; Catalog #CSFLOWBASICA-1) on ice for 2 hours with gentle flicking every 30 min. After washing three times in 1X Bead Wash Buffer (BWB) (Systems Biosciences, CA, United States; Catalog #CSFLOWBASICA-1) on a magnetic stand, the bead/antibody complex was suspended in 400 μL of 1X BWB. 150 μL of EV suspensions were added to the bead/antibody complex and incubated overnight at 4°C with gentle rotation. After EV confirmation by flow cytometry, NEEVs were eluted from the beads using 300 μL of Exosome Elution Buffer (System Biosciences, CA, United States; Catalog #CSFLOWBASICA-1). 150 μL of NEEVs were used immediately for miRNA purification, and the remaining aliquots of NEEVs were stored at -80°C for future analysis.

**2.2.4 Flow Cytometry.** Once the NEEVs were captured and stabilized, the bead/antibody/EV complex was coupled to the EV marker – CD63 fluorescein isothiocyanate (FITC) (CD63-Alexa, Santa Cruz Biotechnology, Fluor 488, SC-5275 AF488, stain concentration 8 µg/ml) and neuronal marker – CD171 Allophycocyanin (APC) (CD171-APC, Invitrogen, REF 17-1719-41, clone eBio5G3 (5G3), stain concentration 0.48 µg/ml) fluorescent tags and subsequently analyzed by flow cytometry to confirm EV capture and NEEV enrichment. The flow cytometric data were acquired at the OU Integrative Immunology Center using an Aurora Flow Cytometer (Cytek Biosciences, Fremont, CA). Instrument performance was validated using cytometer QC beads (Cytek Biosciences, Fremont, CA). FCS data were exported, and analyzed by FlowJo (Tree Star, Inc, Ashland, OR). Debris and small particles were excluded by gating out events with low forward scatter.

**2.2.5 Western Blot.** EVs, NEEVs, EV-depleted plasma, total EV after enrichment, and cells were denatured directly in a 4X Laemmli sample loading buffer and separated by SDS-PAGE using Mini PROTEAN® TGX™ precast gels (Bio-Rad, Catalog # 4561044). The separated proteins were transferred unto polyvinylidene difluoride (PVDF) membranes using a Trans-Blot® Turbo transfer system (Bio-Rad, Catalog # 1704156). PVDF membranes were blocked with 5% non-fat milk powder in Tris-buffered saline containing 0.1% Tween20 (Bio-Rad, Catalog # 1706435) and then were probed with the desired primary antibody overnight at 4°C. Primary antibodies used include CD171 (1:1000, Invitrogen, Catalog # 13-1719-82), CD56 (1:250, Invitrogen, Catalog # MA5-11563), ATP1A3 ( 1:500, US Biological, Catalog # 032268-APC), CD81 (1:1000, Santa Cruz, Catalog # SC-166029), Alix (1:1000, Santa Cruz, Catalog # SC-53540), calnexin (1:1000, Cell Signaling, Catalog # 2679), and APOA1(1:500, Santa Cruz, Catalog # SC-376818). This was followed by incubation with the appropriate peroxidase-conjugated secondary antibody for 1 hour at RT and visualized by Clarity Max Western ECL Substrate (Bio-Rad, Catalog # 1705062). The bands were imaged using ImageQuant LAS 4000 (GE Healthcare Bio-Science, Sweden).

**2.2.6 Particle size and concentration analysis.** The particle concentration and size of EVs and NEEVs were measured using microfluidic resistive pulse sensing (MRPS) with the Spectradyne nCS1^TM^ instrument (Spectradyne Particle Analysis, Signal Hill, CA, USA). The samples were first diluted 1:10 in a 20 nm filtered solution of 1X PBS with 0.05% Poloxamer 188. A small volume of the final solution (5 µL) was analyzed using C-400 cartridges (range from 65 – 400 nm particle size) and the data processed using Spectradyne software.

**2.5.4 Group-level fMRI imaging analysis**

AFNI’s program 3dttest++ was used to assess the whole brain voxel-wise group by NEEV miR-93 interaction on BOLD activation of the VIA interoception versus exteroception contrast. Previous literature has shown that the insula plays an important role in interoception associated with depression^5, 6^; therefore, we performed group analyses using small volume corrections within *a priori* anatomical masks of the insula regions, as well as the whole brain. The resulting group statistical map was corrected for multiple comparisons (within insula, *p* < 0.05; outside of insula p<0.005) using a traditionally conservative approach to avoid the previously stated concerns about false positive rates in fMRI cluster thresholding^7^. A non-Gaussian spatial autocorrelation function (acf) was used in the AFNI programs '3dFWHMx' and '3dClustSim', which estimate intrinsic smoothness and the probability of false positives, respectively. This approach addresses many of the previously existing issues regarding false positives^8^. BOLD activation of the interoception versus exteroception contrast within clusters with significant group*miR-93 effects were extracted for follow-up analyses. Robust regression tested the slope of different relationships between NEEV miR-93 and BOLD for each significant cluster. False Discovery Rate correction for multiple comparisons was used across the resulting tests.

**Supplemental Table S1.** Gene Set Enrichment Analysis (GSEA) biological pathways and genes for hsa.miR-93a-5p

| **Biological Pathways** | **Genes** | **P-value** | **adjusted P-value (BH)** |
| --- | --- | --- | --- |
| GO:0051209_release_of_sequestered_calcium_ion_into_cytosol | PKD2;HTR2A | 0 | 0* |
| GO:0098703_calcium_ion_import_across_plasma_membrane | SLC24A2;TRPV6 | 0 | 0* |
| GO:0008284_positive_regulation_of_cell_population_proliferation | HTR2A;ERBB3;MECP2;FLT1;LIF;EREG;CCND2;FGF4;PTHLH;PURA;PHIP;EIF5A2;CACUL1 | 0.0003 | 0.004* |
| GO:0035725_sodium_ion_transmembrane_transport | PKD2;ATP1A2;ANO6;SCN1A;SLC24A2;SCN2B | 0.0003 | 0.004* |
| GO:0071805_potassium_ion_transmembrane_transport | PKD2;ATP1A2;LRRC55;SLC24A2;KCNK10 | 0.0004 | 0.0042* |
| GO:0031295_T_cell_costimulation | CD274;PDCD1LG2 | 0.0006 | 0.0053* |
| GO:0007613_memory | ATXN1;HTR2A;ATXN1L;SLC24A2;KCNK10 | 0.0017 | 0.0129* |
| GO:0016477_cell_migration | NDEL1;NTNG1;FLT1;RND3;RASGEF1A;ITGB8;ARHGAP35;NANOS1 | 0.0021 | 0.0139* |
| GO:0006816_calcium_ion_transport | PKD2;TRPV6 | 0.0029 | 0.0171* |
| GO:0018108_peptidyl-tyrosine_phosphorylation | ERBB3;ABL2;FLT1;EPHA5;EPHA7;JAK1 | 0.0033 | 0.0175* |
| GO:0070588_calcium_ion_transmembrane_transport | PKD2;ANO6;SLC24A2;TRPV6 | 0.0046 | 0.0222* |
| GO:0006874_cellular_calcium_ion_homeostasis | HTR2A;SLC24A2;STC1 | 0.0086 | 0.0301* |
| GO:0007399_nervous_system_development | ATXN1;ERBB3;C3orf70;TCF4;ARHGAP26;SEMA5A;SCN2B;PURA;PCDHA10 | 0.01 | 0.0301* |
| GO:0010629_negative_regulation_of_gene_expression | ESR1;MECP2;PLAG1;TNRC6C;DIP2A;REST;MYCN;ITGB8 | 0.0098 | 0.0301* |
| GO:0016567_protein_ubiquitination | NEURL1B;NEDD4L;MKRN1;KLHL2;XIAP;MARCHF6;SOCS6;CDC23;FBXL3;GID4;KLHL15 | 0.0075 | 0.0301* |
| GO:0031146_SCF-dependent_proteasomal_ubiquitin-dependent_protein_catabolic_process | FBXO48;FBXO31;FBXL3 | 0.0077 | 0.0301* |
| GO:0042632_cholesterol_homeostasis | LDLR;RORA;LDLRAP1 | 0.0104 | 0.0301* |
| GO:0043547_positive_regulation_of_GTPase_activity | NDEL1;RACGAP1;IQSEC1;ARHGAP26;ARHGAP12;SRGAP1;GNB5;ARHGAP35 | 0.0104 | 0.0301* |
| GO:0051726_regulation_of_cell_cycle | CABLES1;XIAP;MASTL;IRF1;FBXL3 | 0.0108 | 0.0301* |
| GO:0007169_transmembrane_receptor_protein_tyrosine_kinase_signaling_pathway | ERBB3;FLT1;EPHA5;EPHA7 | 0.015 | 0.0398* |
| GO:0007275_multicellular_organism_development | BTBD7;ERBB3;FLT1;LIF;EPHA5;SUCO;EPHA7;PHC3;HLF;WDFY3;GMCL1 | 0.0164 | 0.0414* |
| GO:0051897_positive_regulation_of_protein_kinase_B_signaling | ESR1;ERBB3;PTPRJ;EREG;FGF4;SEMA5A | 0.0197 | 0.0475* |
| GO:0051056_regulation_of_small_GTPase_mediated_signal_transduction | RACGAP1;ARHGAP26;ARHGAP12;SRGAP1;ARHGAP35 | 0.0212 | 0.0489* |

Note. Supplementary Table 1 shows results from gene set enrichment analysis using the miRWalk software. Biological pathways and respective genes are shown. 23 out 53 biological pathways were significant after correction with adjusted p-value <0.05.

**Supplemental Figure S1. Original uncropped blot for illustrative Western Blot used in Figure 1B – CD171.** Well 1: Neuronal-enriched extracellular vesicle (NEEV), Well 2: extracellular vesicles (EV), Well 3: NEEV, Well 4: EV-depleted plasma, Well 5: EV, Well 6: EV supernatant after CD171 enrichment, Well 7: magnetic streptavidin beads after NEEV collection, Well 8: NEEV.

**
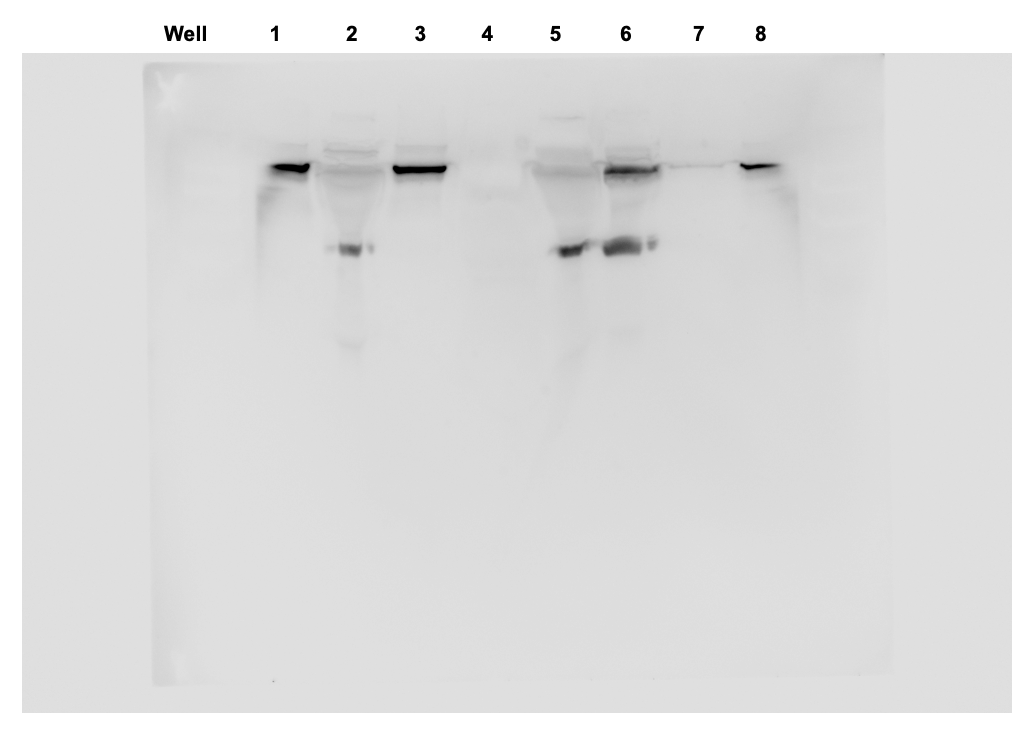
**

**Supplemental Figure S2. Original uncropped blot for illustrative Western Blot used in Figure 1B – CD81.** M: marker; EV: total extracellular vesicles; NEEV: neuronal-enriched extracellular vesicles; SUP1: EV-depleted plasma; SUP2: total EV after enrichment; No EV: PBS instead of total EV were used for enrichment.


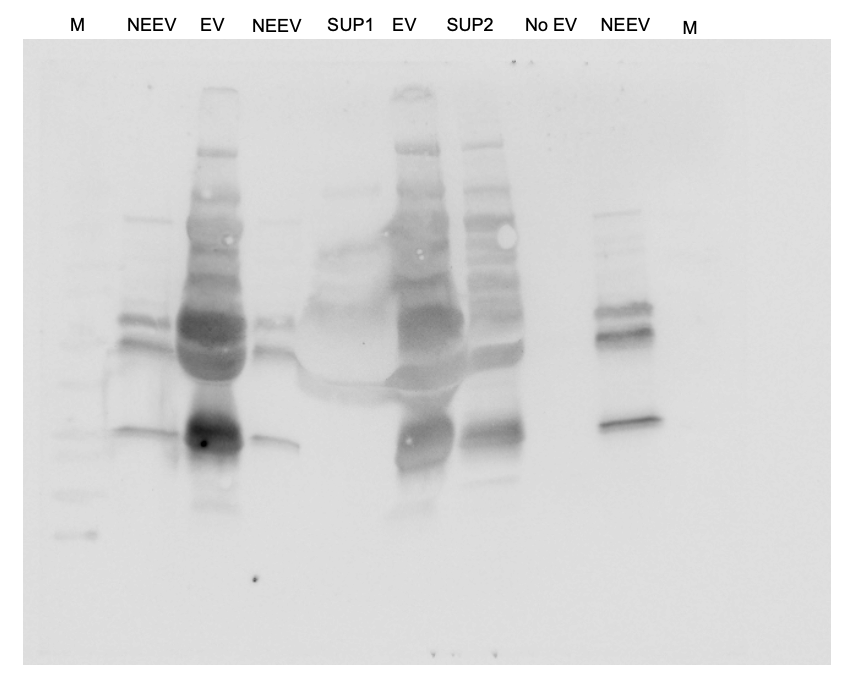


**Supplemental Figure S3. Original uncropped blot for illustrative Western Blot used in Figure 1B – Alix.** M: marker; EV: total extracellular vesicles; NEEV: neuronal-enriched extracellular vesicles; SUP1: EV-depleted plasma; SUP2: total EV after enrichment; No EV: PBS instead of total EV were used for enrichment.

**
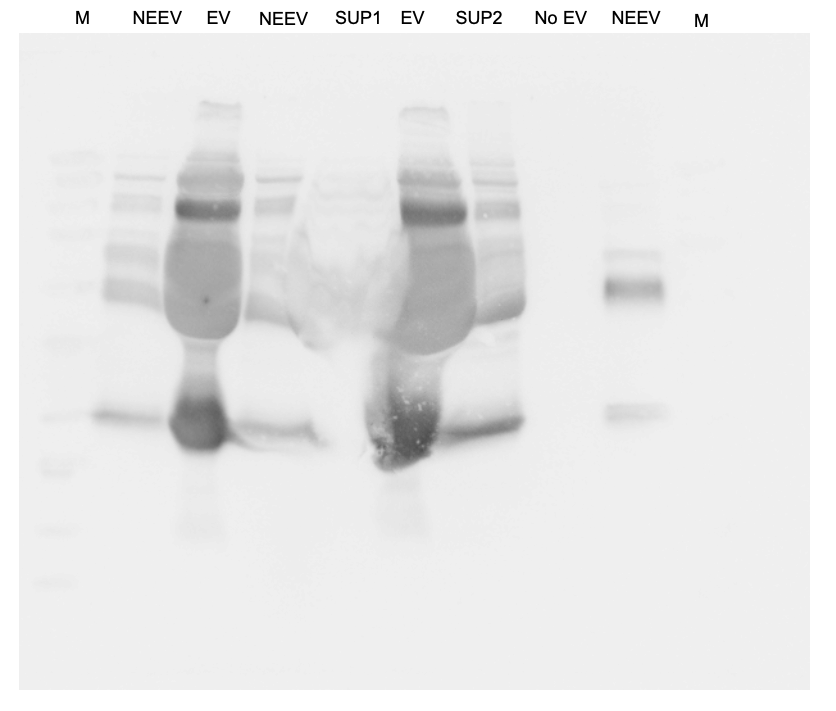
**

**Supplemental Figure S4. Original uncropped blot for illustrative Western Blot used in Figure 1B – Calnexin.** M: marker; EV: total extracellular vesicles; NEEV: neuronal-enriched extracellular vesicles; SUP1: EV-depleted plasma; SUP2: total EV after enrichment; Cells: peripheral blood mononuclear cells.

**
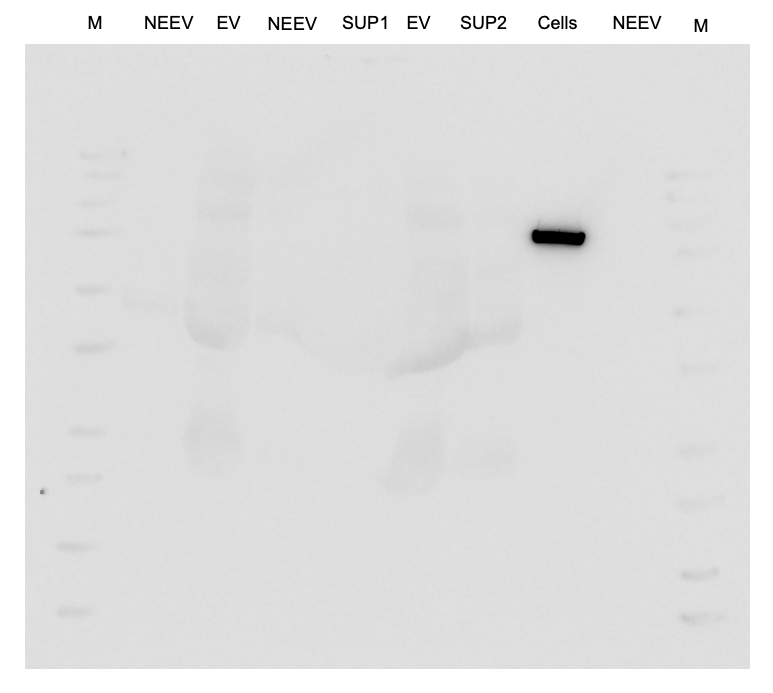
**

**Supplemental Figure S5. Original uncropped blot for illustrative Western Blot used in Figure 1B – NCAM.** Well 1: Neuronal-enriched extracellular vesicle (NEEV), Well 2: No EV: PBS instead of total EV were used for enrichment, Well 3: NEEV.


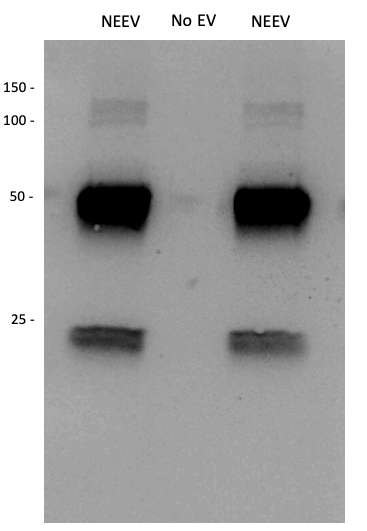


**Supplemental Figure S6. Original uncropped blot for illustrative Western Blot used in Figure 1B – ATP1A3.** Well 1: Neuronal-enriched extracellular vesicle (NEEV), Well 2: No EV: PBS instead of total EV were used for enrichment, Well 3: NEEV.

**
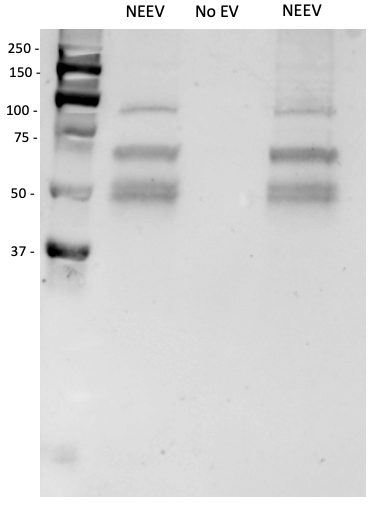
**

**Supplemental Figure S7.** Original uncropped blot for illustrative Western Blot used in Figure 1B – APOA1 Well 1: Neuronal-enriched extracellular vesicle (NEEV), Well 2: Cells: peripheral blood mononuclear cells, Well 3: NEEV.





**NEEV**

**Cells**

**NEEV**

**75 -**

**150 -**

**250-**

**50 -**

**25 -**

**Supplemental Figure S8.** **miR-9 expression in extracellular vesicles (EV) and neuronal-enriched EV (NEEV).**

**
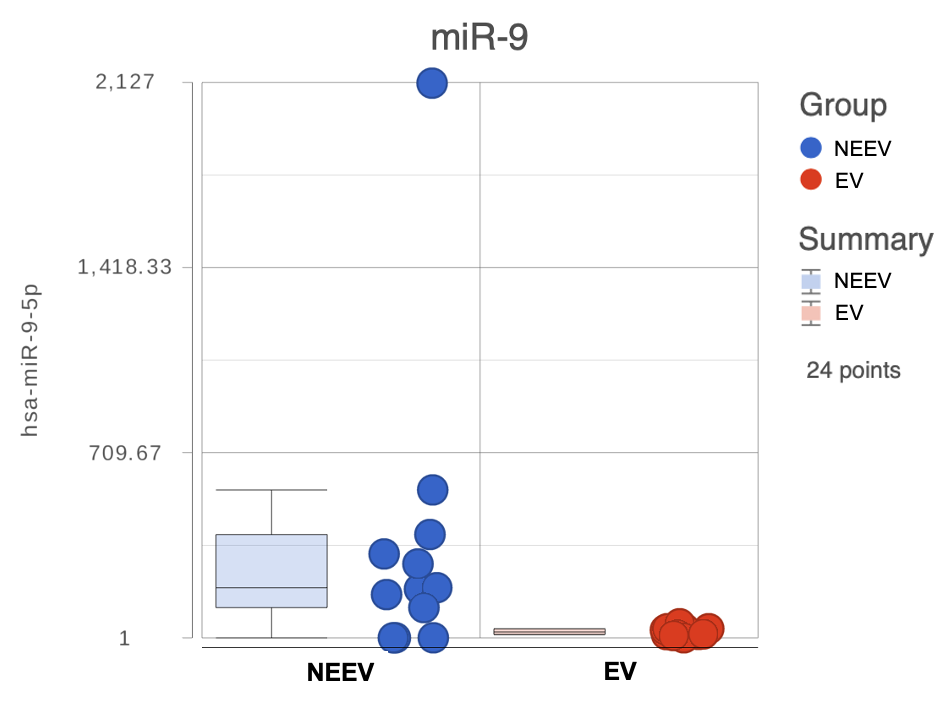
**

**Supplemental Figure S9.** Reactome Pathways for miR-93-5p


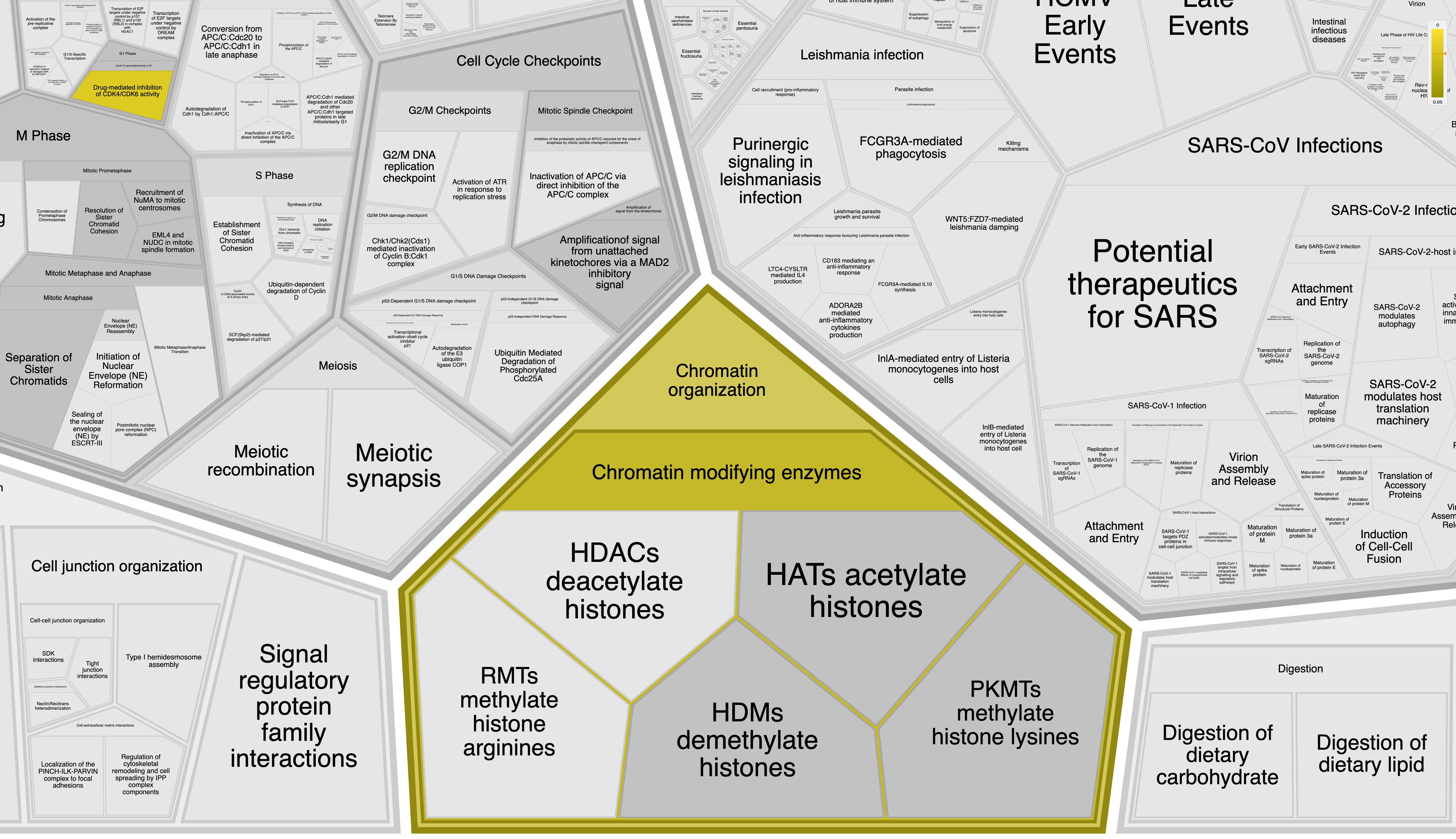


**References**

1. Burrows K, Figueroa-Hall LK, Kuplicki R, Stewart JL, Alarbi AM, Ramesh R *et al.* Neuronally-enriched exosomal microRNA-27b mediates acute effects of ibuprofen on reward-related brain activity in healthy adults: a randomized, placebo-controlled, double-blind trial. *Scientific Reports* 2022; **12**(1).

2. Mustapic M, Eitan E, Werner JK, Jr., Berkowitz ST, Lazaropoulos MP, Tran J *et al.* Plasma Extracellular Vesicles Enriched for Neuronal Origin: A Potential Window into Brain Pathologic Processes. *Front Neurosci* 2017; **11:** 278.

3. Winston CN, Romero HK, Ellisman M, Nauss S, Julovich DA, Conger T *et al.* Assessing Neuronal and Astrocyte Derived Exosomes From Individuals With Mild Traumatic Brain Injury for Markers of Neurodegeneration and Cytotoxic Activity. *Frontiers in Neuroscience* 2019; **13**.

4. Pulliam L, Sun B, Mustapic M, Chawla S, Kapogiannis D. Plasma neuronal exosomes serve as biomarkers of cognitive impairment in HIV infection and Alzheimer's disease. *J Neurovirol* 2019; **25**(5)**:** 702-709.

5. Avery JA, Drevets WC, Moseman SE, Bodurka J, Barcalow JC, Simmons WK. Major depressive disorder is associated with abnormal interoceptive activity and functional connectivity in the insula. *Biol Psychiatry* 2014; **76**(3)**:** 258-266.

6. Barrett LF, Simmons WK. Interoceptive predictions in the brain. *Nat Rev Neurosci* 2015; **16**(7)**:** 419-429.

7. Eklund A, Nichols TE, Knutsson H. Cluster failure: Why fMRI inferences for spatial extent have inflated false-positive rates. *Proc Natl Acad Sci U S A* 2016; **113**(28)**:** 7900-7905.

8. Cox RW, Chen G, Glen DR, Reynolds RC, Taylor PA. fMRI clustering and false-positive rates. *Proc Natl Acad Sci U S A* 2017; **114**(17)**:** E3370-E3371.
